# Introgression of the sesquiterpene biosynthesis from *Solanum habrochaites* to cultivated tomato offers insights into trichome morphology and arthropod resistance

**DOI:** 10.1007/s00425-021-03651-y

**Published:** 2021-06-23

**Authors:** Rodrigo Therezan, Ruy Kortbeek, Eloisa Vendemiatti, Saioa Legarrea, Severino M. de Alencar, Robert C. Schuurink, Petra Bleeker, Lázaro E. P. Peres

**Affiliations:** 1 Department of Biological Sciences, “Luiz de Queiroz” College of Agriculture, Laboratory of Plant Developmental Genetics, University of Sao Paulo, Piracicaba, SP 13418-900 Brazil; 2 Department of Plant Physiology, Green Life Science Research Cluster, Swammerdam Institute for Life Sciences, University of Amsterdam, Science Park 904, 1098 XH Amsterdam, The Netherlands; 3 Molecular and Chemical Ecology, Institute for Biodiversity and Ecosystem Dynamics, University of Amsterdam, PO Box 94240, 1090 GE Amsterdam, The Netherlands; 4 Department of Agri‐Food Industry, Food and Nutrition, “Luiz de Queiroz” College of Agriculture, University of Sao Paulo, Piracicaba, SP 13418-900 Brazil

**Keywords:** Bergamotene, Glandular trichome, Introgressed line, Santalene, Terpenes, Tomato trichome, Piercing-sucking pest

## Abstract

**Main conclusion:**

Cultivated tomatoes harboring the plastid-derived sesquiterpenes from *S. habrochaites* have altered type-VI trichome morphology and unveil additional genetic components necessary for piercing-sucking pest resistance.

**Abstract:**

Arthropod resistance in the tomato wild relative *Solanum habrochaites* LA1777 is linked to specific sesquiterpene biosynthesis. The *Sesquiterpene synthase 2* (*SsT2*) gene cluster on LA1777 chromosome 8 controls plastid-derived sesquiterpene synthesis. The main genes at *SsT2* are *Z-prenyltransferase (zFPS)* and *Santalene and Bergamotene Synthase* (*SBS*), which produce α-santalene, β-bergamotene, and α-bergamotene in LA1777 round-shaped type-VI glandular trichomes. Cultivated tomatoes have mushroom-shaped type-VI trichomes with much smaller glands that contain low levels of monoterpenes and cytosolic-derived sesquiterpenes, not presenting the same pest resistance as in LA1777. We successfully transferred *zFPS* and *SBS* from LA1777 to cultivated tomato (cv. Micro-Tom, MT) by a backcrossing approach. The trichomes of the MT-*Sst2* introgressed line produced high levels of the plastid-derived sesquiterpenes. The type-VI trichome internal storage-cavity size increased in MT-*Sst2*, probably as an effect of the increased amount of sesquiterpenes, although it was not enough to mimic the round-shaped LA1777 trichomes. The presence of high amounts of plastid-derived sesquiterpenes was also not sufficient to confer resistance to various tomato piercing-sucking pests, indicating that the effect of the sesquiterpenes found in the wild *S. habrochaites* can be insect specific. Our results provide for a better understanding of the morphology of *S. habrochaites* type-VI trichomes and paves the way to obtain insect-resistant tomatoes.

**Supplementary Information:**

The online version contains supplementary material available at 10.1007/s00425-021-03651-y.

## Introduction

Terpenoids are the most abundant and diverse class of compounds produced by plants with a wide variety of biological functions (Dudareva et al. 2013). They are produced through combining multiple five-carbon units (C5) of isoprene and are essential for plant growth and development. They participate as precursors for several components of fundamental processes like photosynthesis, respiration, cell-cycle control (Estévez et al. 2001) and plant hormone biosynthesis (Aharoni et al. 2005). Isoprenoids also play an important role in the interactions of plants with the environment, including attraction of pollinators and defense against herbivorous insects (Dudareva et al. 2013; Abbas et al. 2017).

In plants, all isoprenoids originate from two distinct metabolic pathways: the mevalonate (MVA) pathway, located in the cytosol and the 2-C-methyl-D-erythritol 4-phosphate (MEP) pathway located in plastids. Both pathways produce isopentenyl diphosphate (IPP) and dimethylallyl diphosphate (DMAPP). Subsequently, both IPP and DMAPP are converted by prenyltransferases to prenyl diphosphate precursors that are used by terpene synthases (TPSs) to catalyze the formation of C10 monoterpenes, C15 sesquiterpenes, C20 diterpenes and C25 sesterterpenes (Tholl 2006, 2015; Zhou and Pichersky 2020).

In cultivated tomato (*Solanum lycopersicum*), sesquiterpene biosynthesis usually takes place in the cytosol from the MVA pathway. However, in glandular trichomes of the wild tomato species *S. habrochaites,* sesquiterpenes are also produced in the plastids from the MEP pathway (Sallaud et al. 2009). The presence of plastid-derived sesquiterpenes in some wild species has been described to be responsible for the decreased damage by insects, making these wild species naturally resistant to multiple pests, such as lepidopterans (Eigenbrode et al. 1994, 1996), whiteflies (*Bemisia *spp.) (Bleeker et al. 2012) and also spider mites (Maluf et al. 2001).

Two independent loci have been associated with the biosynthesis of two different classes of sesquiterpenes in tomato. The *Sesquiterpene synthase 1* (*SsT1*) locus on chromosome 6 is responsible for the accumulation of cytosol-derived sesquiterpenes. At this locus, *S. lycopersicum SlTPS12* is associated with β-caryophyllene and α-humulene biosynthesis and *SlTPS9* with the production of germacrenes. The *S. habrochaites TPS9* allele (*ShTPS9*) is associated with germacrene B and D production (Hoeven et al. 2000; Bleeker et al. 2011b; Falara et al. 2011). The existence of *SlTPS9*, that makes germacrene C, was also reported for the cultivar VFNT Cherry (Colby et al. 1998), but it is worth noting that this cultivar has introgressions of the wild species *S. peruvianum* on chromosome 6. The second locus harboring sesquiterpenes synthases is the *Sesquiterpene synthase 2* (*SsT2*) on chromosome 8. At this locus *S. habrochaites* has a cluster of *TPSs* genes (*ShTPS18, ShTPS20* and *ShTPS45*) encoding enzymes responsible for the accumulation of plastid-derived sesquiterpenes, including α-santalene, α-bergamotene, β-bergamotene or 7-epizingiberene (Sallaud et al. 2009; Bleeker et al. 2011a). In cultivated tomato, a cluster of five functional *TPS* genes (*SlTPS18*, *SlTPS19*, *SlTPS20*, *SlTPS21*, and *SlTPS41*) is present in the equivalent locus on chromosome 8 (Falara et al. 2011). In addition, this chromosomal region also contains the *Neryl Diphosphate Synthase 1* (*SlNDPS1*) gene, which codes for an enzyme catalyzing the formation of neryl diphosphate (NPP). NPP is used by tomato *SlTPS20* to synthesize β-phellandrene and several other monoterpenes in the plastids of cultivated tomato (Falara et al. 2011; Matsuba et al. 2013). In *S. habrochaites* the *cis-Farnesyl Diphosphate Synthase* (*zFPS*) gene is homologous to the *SlNDPS1* gene (Matsuba et al. 2013). The *zFPS* codes for a *Z*-prenyltransferase that catalyzes the synthesis of *Z-Z-*farnesyl diphosphate (*Z,Z*-FPP) from IPP and DMAPP. The *ShTPS45* gene from *S. habrochaites* LA1777, which is homologous to the tomato *SlTPS20* gene (Bleeker et al. 2012), encodes a *Santalene and Bergamotene Synthase (SBS)* that uses *Z,Z-*FPP as a substrate to produce plastid-derived sesquiterpenes (Sallaud et al. 2009; Matsuba et al. 2013). In *S. habrochaites* PI127826, the *7-epi-zingiberene Synthase (ZIS)* gene, which seems to be allelic to *SBS*, produces the plastid-derived 7-epi-zingiberene (Bleeker et al. 2012). Both *zFPS* and *SBS*, as well as *ZIS*, contain putative chloroplast-targeting sequences allowing the biosynthesis of the sesquiterpenes in this organelle.

The *zFPS* and *SBS* genes are specifically expressed in type-VI glandular trichomes (Sallaud et al. 2009) that are present on several tomato species and wild-relatives (Kang et al. 2010; Glas et al. 2012; Balcke et al. 2017). In this trichome type, metabolites are accumulated inside of intercellular cavity formed between the secretory cells (Tissier et al. 2017). In cultivated tomato, type-VI trichomes are mushroom-shaped containing a single basal cell connected to a short (∼0.1 mm) unicellular stalk which is connected by an intermediate cell to the four-celled glandular head containing chloroplasts and other organelles (Bergau et al. 2015). In *S. habrochaites*, the stalk is longer (∼0.2 mm) and the trichome has a round glandular head instead of 4 visibly distinct cells (Besser et al. 2009). The glandular head of *S. habrochaites* type-VI trichomes has a large intercellular cavity. In contrast, the type-VI trichomes of the cultivated tomato have a very small intercellular cavity, thus leaving little room for the storage of metabolites (Besser et al. 2009). Currently, little is known about the genetic basis of *S. habrochaites* trichome morphology and the impact of an increased storage capacity on insect resistance.

In general, cultivated tomatoes are highly vulnerable to piercing-sucking pests, which include spider mites, thrips and whiteflies. Spider mites and thrips feed on mesophyll cells, whereas whiteflies feed on the phloem sieve tube. Their feeding starts with penetrating their plant hosts, followed by ingesting the cell contents (Freeman et al. 2001; Kindt et al. 2003; Rioja et al. 2017). In addition, whiteflies and thrips can spread viruses (Jones 2005; Moodley et al. 2019). Under heavy infestation, these pests can cause a reduction of plant vigor and yield which can lead to huge losses in productivity (Wakil et al. 2018). Consequently, to minimize the damage caused by pests, high amounts of pesticides have been applied in agri- and horticulture (Silva et al. 2011). In this sense, an alternative to chemical pest control could be the use of commercial tomatoes carrying favorable genetic factors from tomato wild species. Herein, we investigated whether the introduction of the genetic pathway for plastid-derived sesquiterpenes from a wild species into cultivated tomato could increase resistance to piercing-sucking tomato pests. We show that the *SsT2* gene cluster that controls α-santalene, α-bergamotene and β-bergamotene production in *S. habrochaites* LA1777 can be effectively transferred to cultivated tomato (cv. Micro-Tom) and functions in its type-VI trichomes. Tomato type-VI trichomes that accumulated high levels of plastid-derived sesquiterpenes also increased the size of the internal gland cavity, providing a better understanding of differential trichome morphology. We further demonstrated that the production of “wild tomato sesquiterpenes” was not sufficient to confer resistance to the piercing-sucking tomato pests tested. Therefore, additional genetic factors outside the metabolic cluster on chromosome 8 are required for the production of the anti-insect compounds in *S. habrochaites* LA1777 that might affect piercing-sucking pests.

## Materials and methods

### Plant material and growth conditions

Seeds from Micro-Tom (MT) were donated by Dr. Avram Levy (Weizmann Institute of Science, Israel) in 1998 and maintained through self-pollination as a true-to-type cultivar since then. The *lutescent 1* mutation (Solyc08g005010) (Liu et al. 2021) was introgressed into MT from its original background as described (Carvalho et al. 2011). Seeds from *Solanum habrochaites* LA1777 were obtained from the Tomato Genetics Resource Center (TGRC—University of California).

**Fig. 1:**
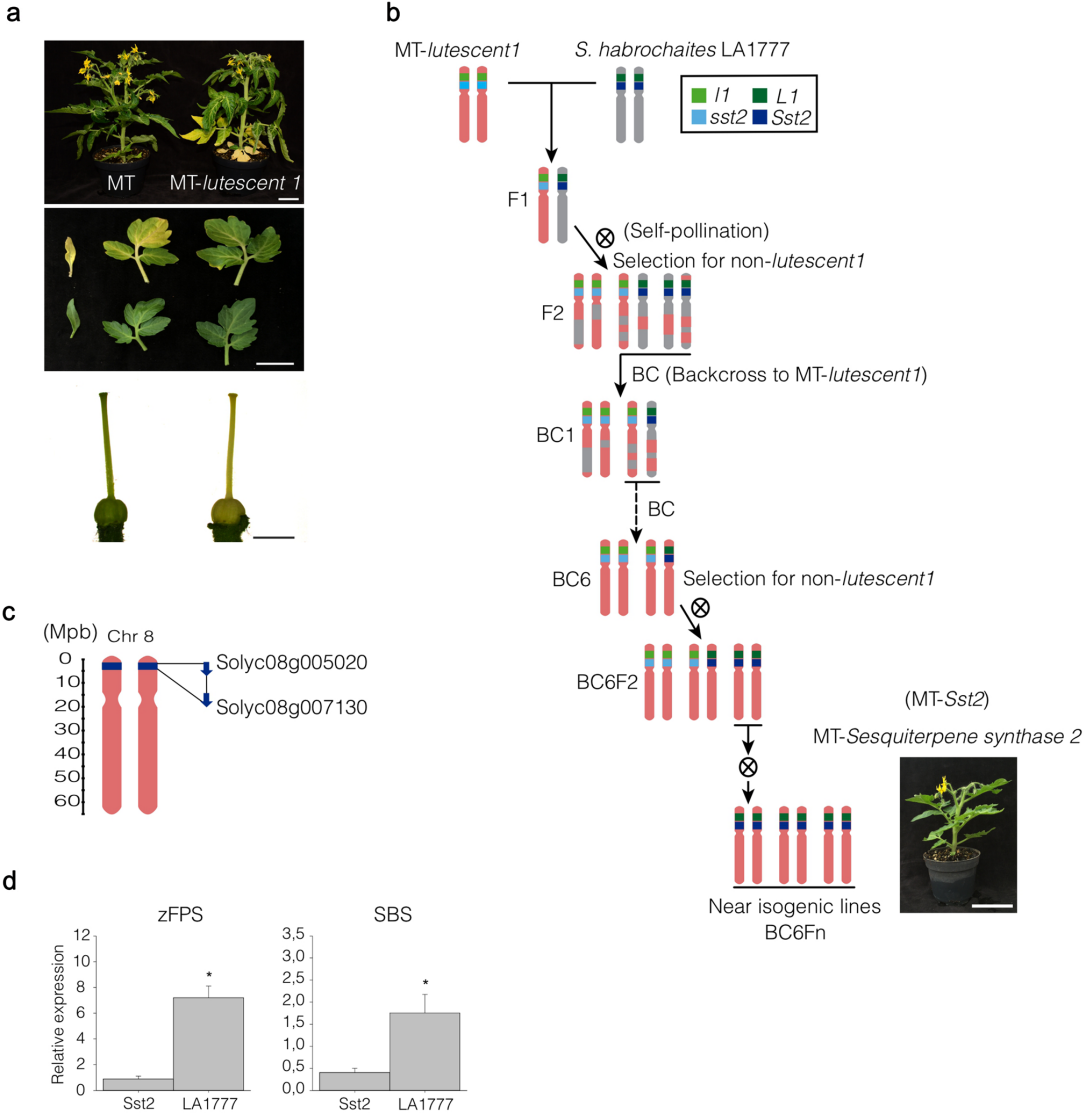
**a** Representative MT and MT near-isogenic line (NIL) harboring the lutescent 1 (l/l) mutation. b Scheme of crossing and backcrossing (BC) to create a Micro-Tom (MT) near-isogenic line (NIL) harboring the Solanum habrochaites LA1777 genes for the “Sesquiterpene Synthase 2” (SsT2) locus. MT NIL bearing the lutescent 1 mutation was used to assist the introgression process as a morphological marker (the absence of the lutescent 1 phenotype was used as an indicator of the presence of the LA1777 genes in the SsT2 locus). The presence of MT (l1 and sst2) or LA1777 (L1 and Sst2) variants is indicated in different colors. c The ID (Solyc) of the genetic markers used to determine the introgression borders are depicted. d Transcript levels of the SsT2 locus-derived genes cis-Farnesyl Diphosphate Synthase (zFPS) and Santalene and Bergamotene Synthase (SBS) in trichomes from MT-Sst2 and S. habrochaites LA1777. The expression of both zFPS and SBS in MT-Sst2 genotype, although lower than that of LA1777, evidences the introgression of the targeted chromosomal segment. Mean values of 4 biological replicates are shown. Transcript levels were normalized for Rubisco conjugating enzyme 1 (RCE1). Asterisks indicate mean significantly different from MT-Sst2, according to Student’s t test (P  ≤  0.05)

The sesquiterpene synthase 2 pathway from *S. habrochaites* LA1777 was introgressed into MT background by allelic substitution making use of the morphological marker MT-*lutescent 1*, which maps on the same arm of chromosome 8 (https://tgrc.ucdavis.edu/) (Fig. 1a, b) (Liu et al. 2021). Briefly, pollen from *S. habrochaites* LA1777 were collected and used to fertilize emasculated MT-*lutescent 1* flowers. The F_1_ obtained was used as pollen donor for MT-*lutescent 1* plants and this procedure was repeated in the successive backcrossings (BC). In each BC, we screened for reduced plant size (MT-like phenotype) and the absence of the *lutescent 1* phenotype (Fig. 1a), which is indicative for the presence of the LA1777 genes in the *SsT2* locus. After self-pollination in BC_6_F_2_ generation, we screened plants for the presence of the same sesquiterpenes compounds found in the wild parental species. The resulting homozygous MT-*Sst2* genotype was considered a near-isogenic line (NIL).

Plants were grown in a greenhouse with 30/26 °C temperature day/night and 60–75% ambient relative humidity, 11.5 h/13 h (winter/summer) photoperiod, sunlight 250–350 μmol photons m^−2^ s^−1^ PAR irradiance. Seeds were germinated in bulk in 350 mL pots with a 1:1 mixture of commercial potting mix Basaplant^®^ and expanded vermiculite and supplemented with 1 g L^−1^ 10:10:10 NPK and 4 g L^−1^ dolomite limestone (MgCO_3_ + CaCO_3_). Upon the appearance of the first true leaf, seedlings of each genotype were individually transplanted to 150 mL pots containing the soil mix described above, except that NPK supplementation was increased to 8 g L^−1^.

### Genetic and Physical Mapping of the introgressed *Sst2* genes

Genomic DNA isolation was extracted from leaflets using the method described (Fulton et al. 1995). Molecular mapping using cleaved amplified polymorphic sequence (CAPS) markers was performed as previously described (Shavrukov 2016). Details of tomato genetic maps and chromosome 8 molecular markers can be accessed through the Solanaceous Genomics Network (http://solgenomics.net/). Primers and restriction enzymes yielding CAPS between tomato and *S. habrochaites* LA1777 are detailed in Supplemental Table S1.

### Trichome counts and phenotyping

Counting of trichomes density (mm^2^) was performed on leaflets taken from mature fifth leaves (counting from the cotyledons) according to the methodology described (Vendemiatti et al. 2017). Both leaf surfaces were dissected along the longitudinal axis in 15 × 3 mm strips covering the middle section of the leaf blade (avoiding the primary veins). The strips were fixed on microscope slides using transparent nail polish. Five individuals per genotype were sampled, and four different strips were analyzed per plant. Images were taken using a Leica S8AP0 stereomicroscope (Wetzlar, Germany) magnifying glass set to 50 × magnification, coupled to a Leica DFC295 camera (Wetzlar, Germany).

The morphology of type-VI trichomes was examined under an EVOSfl (www.thermofisher.com) inverted microscope. Lateral leaflets strips were submerged in water under microscope slides and images of type-VI trichomes were taken (Fig. 4a). All trichome measurements (stalk length, gland and cavity axis) were performed on images of 5 plants per genotype using ImageJ software version 1.4.1. Gland and cavity volume were calculated using the volume of the ellipsoid formula: $V\, = \,{4/3}\, \times \,{\uppi }\, \times \,a^{{2}} \left( {\text{horizontal radius}} \right)\, \times \,c\left( {\text{vertical radius}} \right)$.

### GC–MS quantification

For GC–MS volatile terpene quantification, 300 individual type-VI trichome glands were collected from leaves in adult vegetative phase (fifth leaf from the cotyledons) with a glass pulled Pasteur pipette under a Leica MZFLIII microscope (www.leica-microsystems.com). The terpene extraction was conducted according to the methodology described (Xu et al. 2018c). The collected glands were dissolved in 150 µL of hexane plus 0.5 ng/µL of benzyl acetate (Sigma-Aldrich; www.sigmaaldrich.com) as an internal standard. Na_2_CO_3_ (Sigma-Aldrich) was used to remove water from the hexane. Volatiles were separated using an Agilent (www.agilent.com) 7890A gas chromatograph, attached to an Agilent 7200 accurate-mass quadrupole time-of-flight mass spectrometer. Here, 2 µL of the sample was injected, heated to 275 °C in the injector port and separated on a HP-5 ms column (0.25 mm in diameter, 30 m in length, with 0.25 μm film thickness) using Helium as carrier gas (flow rate 1 mL/min). The oven temperature was maintained at 40 °C for 3 min and increased by 15 °C per min until it reached 250 °C and maintained for 3 min. Identification of the compounds was based on the retention time of the chromatographic peaks and their corresponding mass spectra, which were compared to terpene standards and data libraries. Quantification of peak areas was performed using Masshunter Qualitative Analysis software (Agilent). Peak areas were corrected for the internal standard and quantified using the available terpene standards. For compounds without terpene standards, we used β-caryophyllene standard as a reference. Terpene concentration was calculated per trichome gland (ng/gland) using the peak areas relative to the internal (benzyl acetate) and terpene standards available.

### Whitefly bioassay

*Bemisia tabaci* (former biotype B; Middle East Asia Minor I-II (MEAM)) population was maintained in a climatized chamber (Snijders Tilburg; T 28 °C, 16-h light, RH 75%) on cucumber plants prior to the experiment. For no-choice assays, twenty adult whiteflies were randomly taken from the population, anesthetized with CO_2_ and placed in a clipcage (2.5 cm diameter; Bioquip). Two clipcages were attached to two different leaflets per plant. Five plants per genotype were used. The plants were kept inside of a closed greenhouse compartment (28 °C, RH 65%) and after 5 days, the number of whiteflies alive was recorded.

### Spider mite bioassay

A no-choice performance assay was set up using two species of spider mites. The two spotted spider mite *T. urticae* Koch Viçosa-1 and the red spider mite *T. evansi* Baker and Pritchard Viçosa-1 were initially collected from infested tomato plants (Sarmento et al. 2011). Before the experiments, *T. urticae* mites were maintained on detached leaves of *S. lycopersicum* cv. Santa Clara and *T. evansi* mites were maintained on detached leaves of *S. lycopersicum* cv. Castlemart following standard procedures (Ataide et al. 2016). The rearings were maintained in a climate room at 25 °C, a 16/8 h light regime with 300 µE m^−2^ s^−1^, and 60% RH.

For each plant genotype, 15 leaf disks of 15 mm were made from the fifth leaf (counting from the cotyledons). The leaf disks were placed on 1.5% Daishin agar (Duchefa Biochemie, Haarlem, The Netherlands) that was poured in small cups (3 cm diameter  ×  2 cm height) with their adaxial side facing up. On each leaf disk, a single 2-day-old adult female of *T. urticae* or *T. evansi* was placed using a soft paintbrush. Mites were confined into each cup and ventilation was assured by a 1 cm^2^ opening on the lid that was covered with mite-proof mesh (pore size of 80 µm). The cups were maintained in a climate room at 25 °C, a 16/8 h light regime with 300 µE m^−2^ s^−1^, and 60% RH. Two days after infestation, spider mite survival was recorded, and the average fecundity (number of eggs laid per female) was calculated using those spider mites that were alive after the 2-day period for the calculations.

### Thrips bioassay

A no-choice performance thrips bioassay was set up using the western flower thrips *Frankliniella occidentalis* (Pergande)*.* The thrips colony was kept in the laboratory inside cages where bean pods were provided and supplemented with pollen as previously described (Muñoz-Cárdenas et al. 2017). For each plant genotype, 15 leaf disks of 15 mm diameter were made. Similar to the spider mite set-up, the experimental arena consisted of cups (3 cm diameter  ×  2 cm height) filled with 1.5% Daishin agar on which one leaflet was placed with the adaxial side up. Five adult females were collected from the colony with the help of a 1 ml pipette tip attached to a vacuum and were released inside each cup through a small opening on the side of the cup, that was otherwise sealed with parafilm. The cups were maintained in a climate room at 25 °C, a 16/8 h light regime with 300 µE m^−2^ s^−1^, and 60% RH. 72 h after the release of the females, the adult thrips were removed, and their survival (number of alive thrips) was scored with the help of a dissecting stereoscope. The number of larvae that emerged from the eggs laid during the experiment was assessed 7 days after the beginning of the experiment.

### RNA isolation and quantitative RT-PCR

Total RNA was extracted from trichomes isolated by shaking stems in liquid nitrogen with a vortex mixer. Total RNA was isolated using Trizol reagent (Invitrogen) according to the manufacturer’s instructions. RNA treated with TURBO DNase (Ambion; www.thermofisher.com) was reverse-transcribed to generate first-strand cDNA using RevertAid H Minus Reverse Transcriptase (Fermentas; www.thermofisher.com). cDNA was used as a template for quantitative RT-PCR (qRT-PCR). PCR reactions were performed using HOT FIREPol EvaGreen qPCR Mix Plus (Solis Biodyne; www.sbd.ee) and analyzed in an ABI 7500 Real-Time PCR System (Applied Biosystems; www.appliedbiosystems.com). Two technical replicates were analyzed for at less three biological samples, together with template free reactions as negative controls. Transcript abundances were normalized to *Rubisco conjugating enzyme 1* (*RCE1*) expression. Detailed primers information is described in the Supplemental Table S2.

### Experimental design and statistical analysis

Statistical analyses were done using SigmaPlot 11.0 for Windows. The experiments were arranged in a completely randomized design. All data were tested for normality and equal variance by Kolmogorov–Smirnov tests. The means were further analyzed by two-tailed Student’s *t *test (*P * ≤  0.05) or Fisher’s LSD test (*P*  ≤  0.05) after one-way ANOVA in multiple comparisons. For data that do not assume a specified variance or normality, we performed ranking tests Wilcoxon rank-sum for pairwise comparisons and Kruskal–Wallis one-way analysis for multiple groups.

## Results

### Introgression of *SsT2* gene cluster from *S. habrochaites* LA1777 into *S. lycopersicum* cv. Micro-Tom (MT)

To introduce the plastid-derived sesquiterpene pathway into the Micro-Tom (MT) cultivar, we crossed *S. habrochaites* LA1777 with a MT line harboring the *lutescent 1* mutation (MT-*l*1) and used it as a recurrent parental (Fig. 1a, b). Both the *lutescent 1* mutation (Liu et al. 2021) and the *SsT2* locus map at the short arm of the chromosome 8 (Tanksley et al. 1992; Sallaud et al. 2009). The *lutescent 1* phenotype comprises a premature and progressive yellowing of the leaves due to impaired chlorophyll accumulation (starting from the base of the plant) (Fig. 1a), a lack of chlorophyll accumulation in the pistils (Fig. 1a) and whitish-yellow fruits (Barry et al. 2012). This allowed us to introgress the *S. habrochaites SsT2* locus into MT by a relatively easy visual selection of the progeny. In each generation of introgression, we selected for plants not presenting the *lutescent 1* phenotype, which implies the presence of the equivalent chromosome segment of *S. habrochaites* containing the *SsT2* locus. In the F_2_ generation, plants not harboring the *lutescent 1* mutation were used for backcrossing (BC) with MT-*l1*. This procedure was repeated in the BC_1_ and subsequent generations. After six BC generations and self-pollination (BC_6_F_2_) using the visual marker, we employed CAPS markers based on single-nucleotide polymorphisms (SNPs) for a genetic screen for homozygous plants harboring the *SsT2* locus from the wild species. In the BC_6_F_3_ generation and generations thereafter (BC_6_F_n_), the obtained plants were considered a near-isogenic line (NIL), no longer segregating for the presence of the wild sesquiterpenes pathway and other traits. The NIL was named MT-*Sesquiterpene synthase 2* (MT-*Sst2*).

We employed CAPS markers to determine the size of the fragment introgressed into the MT background. Genetic mapping positioned the introgressed region between *Solyc08g005020* and *Solyc08g007130* on the top of chromosome 8 (Fig. 1c). The introgressed region overlaps with the mapping position of *SsT2* previously reported (Sallaud et al. 2009), which also confirmed that both *zFPS* and *SBS* genes were mapped to this region, and it is furthermore consistent with the *SsT2* locus described (Hoeven et al. 2000). It is worth noting, however, that the genotype used by Hoeven et al. (2000) and Sallaud et al. (2009) contain more than one chromosomal segment introgressed. The NIL MT-*Sst2* produced here is more adequate for comparative studies since it has only a relatively small chromosomal segment introgressed in chromosome 8.

Additionally, we analyzed the relative transcript levels of *zFPS* and *SBS* by quantitative RT-PCR in trichomes of MT-*Sst2* and *S. habrochaites* LA1777. Both genes were indeed expressed in MT-*Sst2*, but transcripts levels were significantly lower in MT-*Sst2* compared to the wild parental (Fig. 1d).

### Production of the sesquiterpenes α-santalene, α-bergamotene and β-bergamotene in the Micro-Tom line harboring the *S. habrochaites SsT2* locus

**Fig. 2:**
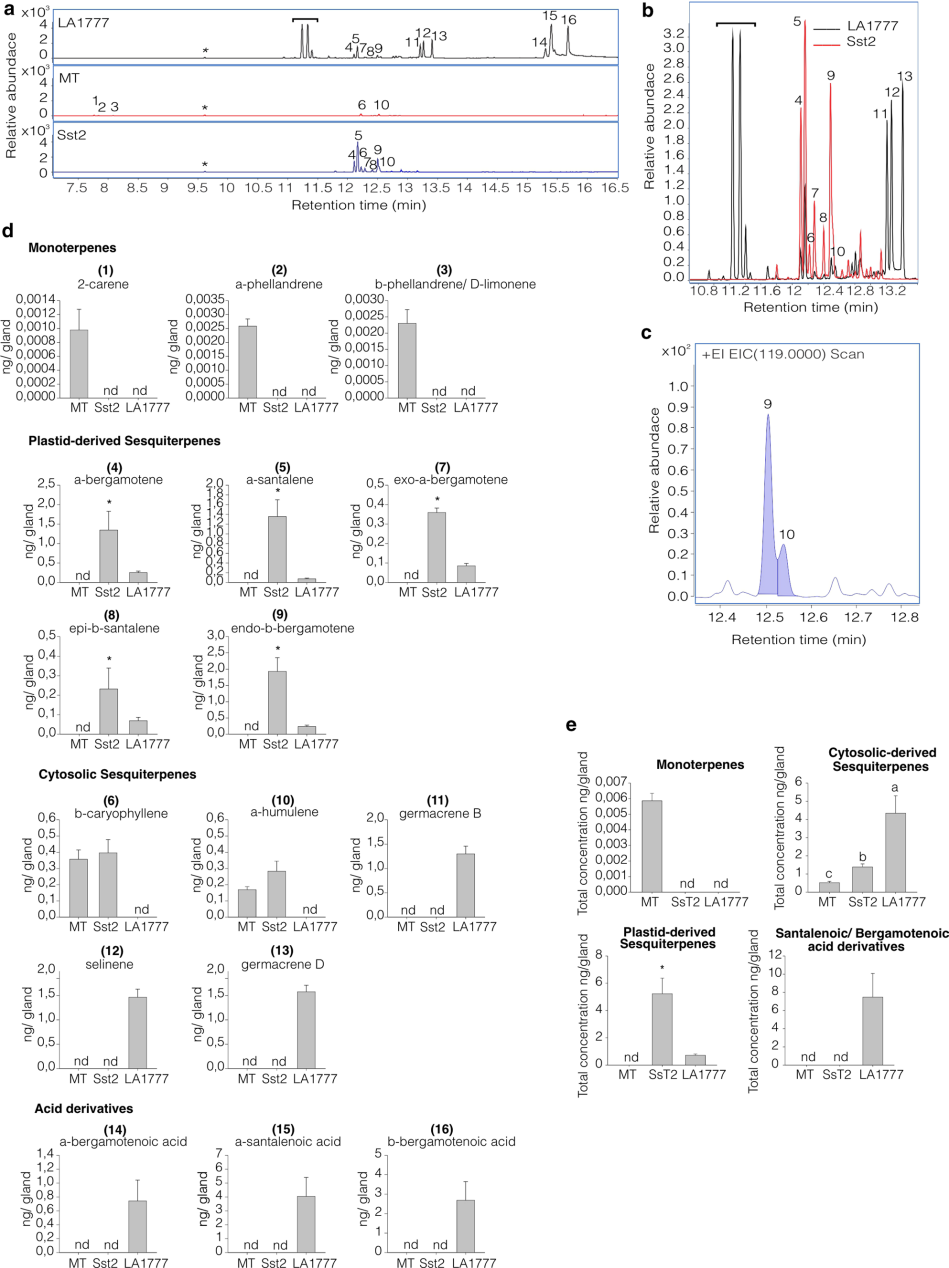
**a** GC–MS chromatograms showing mono and sesquiterpenes found in type-VI trichomes from Micro-Tom (MT), MT-*Sst2,* and *S. habrochaites* LA1777. The indicated peaks correspond to the following compounds: (1) 2-carene, (2) α-phellandrene, (3) β-phellandrene/D-limonene, (4) α-bergamotene, (5) α-santalene, (6) β-caryophyllene, (7) exo-α-bergamotene, (8) epi-β-santalene, (9) endo-β-bergamotene, (10) α-humulene, (11) germacrene B, (12) selinene, (13) germacrene D (14) α-bergamotenoic acid, (15) α-santalenoic acid and (16) β-bergamotenoic acid. The asterisks indicate the peaks related to the internal standard. The bracket indicates peaks related to unidentified putatively lipid-originating compounds. The chromatogram shows the detector response for ion mass 93.069 and 108.056. **b** Gas chromatogram overlaying sesquiterpenes found in type-VI trichomes from MT-*Sst2* and *Solanum habrochaites* LA1777. **c** Gas chromatogram showing (9) endo-β-bergamotene and (10) α-humulene peaks from MT-*Sst2* plants. Chromatogram shows the detector response for ion mass 119.000. **d** Amount of compounds presents in type-VI glandular trichomes of each genotype. Concentration of monoterpenes: (1) 2-carene, (2) α-phellandrene and (3) β-phellandrene/D-limonene; Total concentration of cytosolic sesquiterpenes: (6) β-caryophyllene, (10) α-humulene, (11) germacrene B, (12) selinene and (13) germacrene D; Concentration of plastid-derived sesquiterpenes: (4) α-bergamotene, (5) α-santalene, (7) exo-α-bergamotene, (8) epi-β-santalene and (9) endo-β-bergamotene; Concentration of santalenoic/bergamotenoic acid derivative: (14) α-bergamotenoic acid, (15) α-santalenoic acid (16) β-bergamotenoic acid. **e** Total amount of compounds presents in type-VI glandular trichomes of each genotype. The bars represent the mean  ±  SE of five biological replicates. For each sample, 300 type-VI glandular trichomes were collected with a glass capillary for GC–MS analysis. Bars indicated with an asterisk were significantly different according to *t* test (*P*  ≤  0.05). Bars indicated with different letters were significantly different according to Fisher’s LSD test (*P*  ≤  0.05) after ANOVA. *nd* not detected

To confirm the presence of the *S. habrochaites* sesquiterpene pathway in the MT-*Sst2* line, we performed Gas-chromatography Mass-Spectrometry (GC–MS) analyses on isolated type-VI trichome glands. The type-VI trichomes of the MT-*Sst2* line not only accumulated α-santalene, α-bergamotene and β-bergamotene, but also produced higher amounts of these specific compounds, compared to the parental *S. habrochaites* LA1777 (Fig. 2a–d, peaks 4, 5, 7, 8 and 9).

As expected, the monoterpenes found in the MT parental (2-carene, α-phellandrene and β-phellandrene and D-limonene) were not detected in LA1777 and in the MT-*Sst2* line (Fig. 2a–d, peaks 1, 2 and 3). Since these plastid-derived monoterpenes are associated to the presence of the *SlTPS20* genes on *S. lycopersicum* chromosome 8, it was predicted that homozygous MT-*Sst2* plants would replace them by the plastid-derived sesquiterpenes coded by *S. habrochaites* *TPS* genes introgressed on the same chromosomal region. Indeed, the MT-*Sst2* line produced the plastid-derived sesquiterpenes, α-bergamotene, α-santalene, exo-α-bergamotene, epi-β-santalene, and endo-β-bergamotene, also present in *S. habrochaites* LA1777 but not in MT (Fig. 2a–d). Although we found high levels of plastid-derived sesquiterpenes in the MT-*Sst2* line, we did not detect any santalenoic and bergamotenoic acids derivatives, as in the wild species (Fig. 2a–d, peaks 14–16). As expected, no α-santalene, α-bergamotene, β-bergamotene and their acid derivatives were present in control MT trichomes (Fig. 2a–e).

We also noted that the concentration of the cytosolic-derived sesquiterpene α-humulene was higher in MT-*Sst2* when compared to the control MT. The TPS12 responsible for the production of β-caryophyllene and α-humulene is encoded by a gene in the *SsT1* locus on chromosome 6, which is outside of the region introgressed. Therefore, the *TPS12* in the *SsT1* locus is likely to have the same alleles for both genotypes and it could not be the cause of the higher amount of α-humulene in MT-*Sst2*. Besides, we did not find the same increase in β-caryophyllene. Since α-humulene coelutes with endo-β-bergamotene (Hoeven et al. 2000) (Fig. 2a, peaks 9 and 10), we tried to improve the separation of both endo-β-bergamotene and α-humulene. After our attempt we still found a small overlap of α-humulene with peak 9 (endo-β-bergamotene) (Fig. 2c, peaks 9 and 10), but the separation was sufficient to distinguish endo-β-bergamotene from α-humulene. Consequently, β-caryophyllene and α-humulene in MT-*Sst2* did not differ statistically from MT plants.

### Production of mono and sesquiterpenes in type-VI trichomes under different allelic dosages at the *SsT2* locus

To investigate how the allelic dosage at the *SsT2* locus affects the levels of mono and sesquiterpenes in type-VI trichomes, we compared homozygous *Sst2*/*Sst2*, heterozygous *sst2*/*Sst2* plants, as well as the control MT (*sst2*/*sst2)*. The molecular markers used for genotype selection were designed based on polymorphisms found in the genomic sequence of cultivated tomato and LA1777 (Table S1). Note that there is no complete synteny between the *SsT2* locus of *S. lycopersicum*, and *S. habrochaites.* Hence, at this locus, *S. lycopersicum* contains the functional genes *SlTPS18*, *SlTPS19*, *SlTPS20*, *SlTPS21*, *SlTPS41* and *SlNDPS1*, whereas *S. habrochaites* contains the functional genes *ShTPS18*, *ShTPS20*, *ShTPS45* (*SBS*) and *zFPS* (Matsuba et al. 2013). Thus, the *sst2/Sst2* plants can be better considered hemizygous for both sets of genes from each parental.

**Fig. 3:**
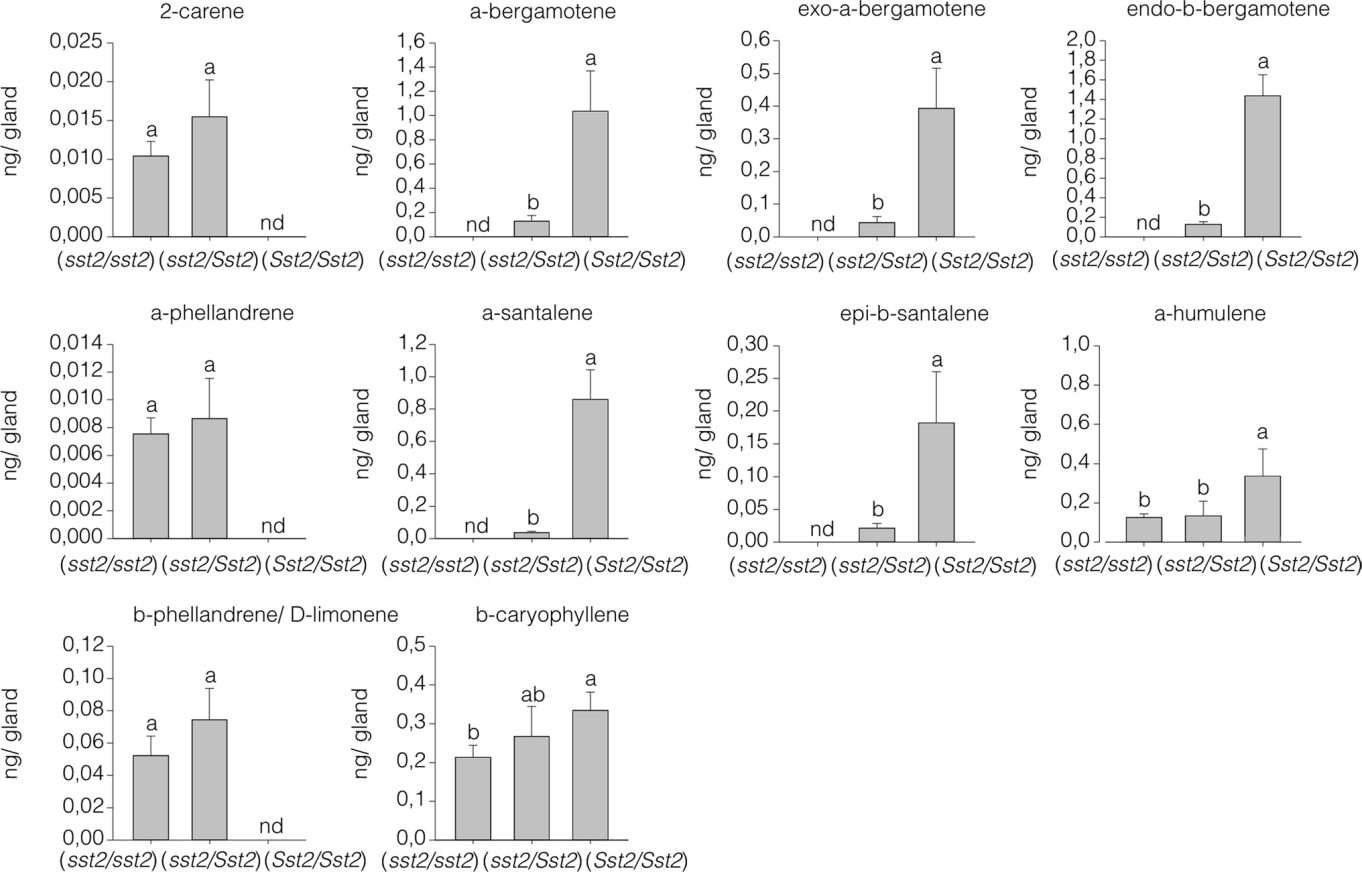
Volatile terpene levels in type-VI glandular trichomes from control Micro-Tom (*sst2/sst2*) and near-isogenic lines homozygous (*Sst2/Sst2*) and hemizygous (*sst2/Sst2*) for *S. habrochaites* alleles at the *SsT2* locus. The data show the amount of each compound present in type-VI glandular trichomes. Each data point represents the mean and SE of five biological replicates. For each sample, 300 type-VI glandular trichomes were collected with a glass capillary before GC–MS. Bars indicated with different letters were significantly different according to Fisher’s LSD test (*P*  ≤  0.05) after ANOVA. *nd* not detected

As predicted, the monoterpenes 2-carene, α-phellandrene and β-phellandrene and D-limonene were absent in the type-VI trichomes of homozygous *Sst2/Sst2* plants, since they lack the *SlNDPS1* and *SlTPS20* genes due to the introgression of the *S. habrochaites* chromosomal segment correspondent to the *SsT2* locus. There was no significant effect of allelic dosage for *SlNDPS1* and *SlTPS20* when comparing monoterpene content of the hemizygous *sst2/Sst2* plants and the homozygous (*sst2/sst2*) MT control (Fig. 3). Regarding the concentration of cytosolic-derived sesquiterpenes β-caryophyllene and α-humulene, which are encoded by the *SsT1* locus, the hemizygous *sst2/Sst2* plants did not differ from the control MT (*sst2/sst2)* (Fig. 3).

The concentration of all plastid-derived sesquiterpenes α-bergamotene, α-santalene, exo-α-bergamotene, epi-β-santalene, and endo-β-bergamotene was higher in the homozygous *Sst2*/*Sst2* type-VI trichomes when compared to the trichomes of hemizygous *sst2*/*Sst2* plants, indicating that these compounds are under the effect of allelic dosage (Fig. 3).

### Trichome abundance and morphology in the Micro-Tom line harboring the *S. habrochaites* genes in the *SsT2* locus

**Fig. 4:**
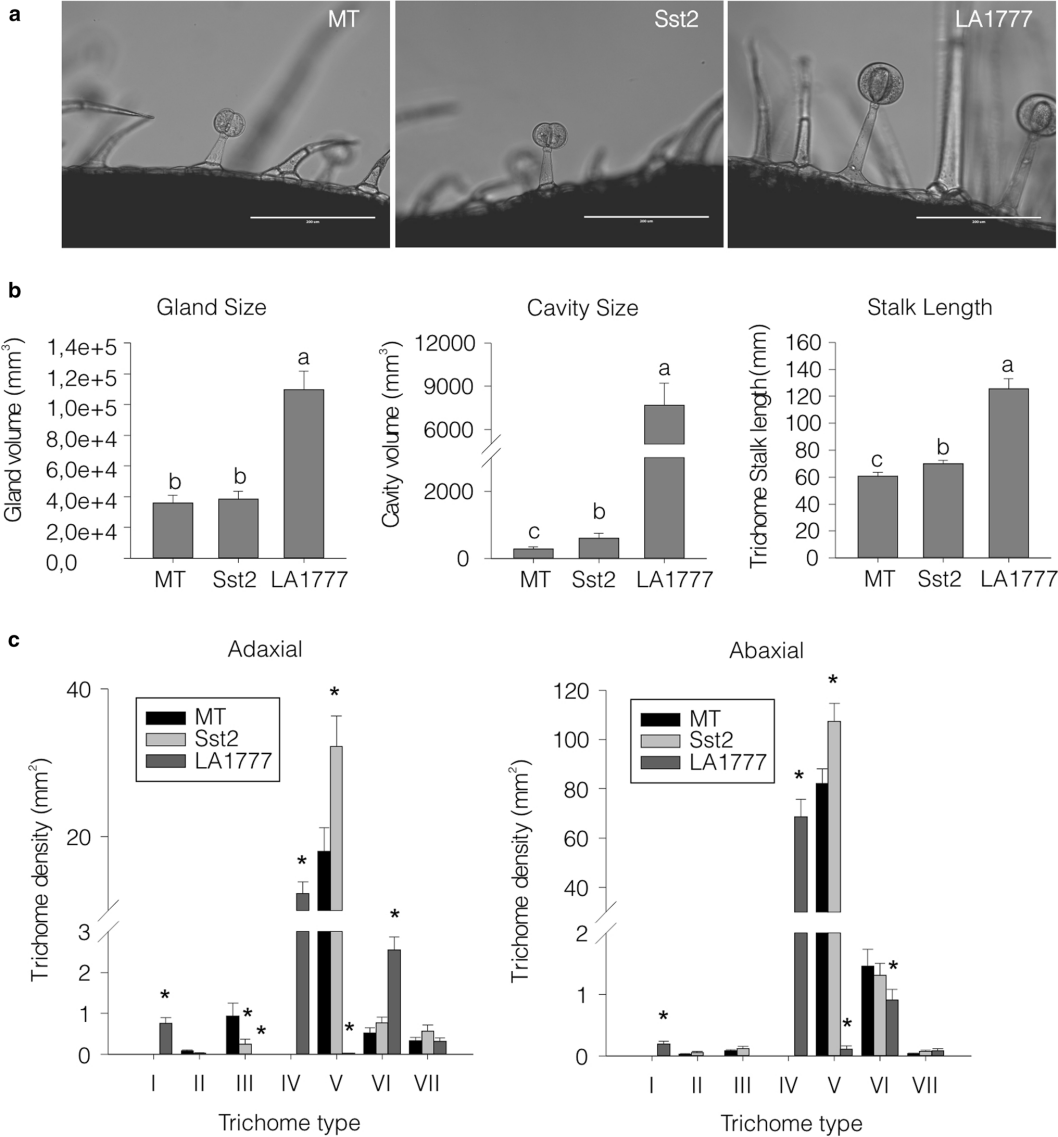
**a** Bright field microscopy of trichomes on the leaf surface of representative 45-days old plants of Micro-Tom (MT), MT-*Sst2*, and *Solanum habrochaites* LA1777. Scale bar  =  200 μm. **b** Trichome gland size, cavity volume and stalk length of type-VI trichomes. Data are mean (±  SE) of 20 trichomes of two replicate leaves of five plants. Bars indicated with different letters were significantly different according to Fisher’s LSD test (*P*  ≤  0.05) after ANOVA. **c** Density (mm^2^) of trichome types on adaxial and abaxial leaf surfaces. Data are mean (*n * =  40) for each surface. Asterisks indicate mean significantly different from the control MT, according to Student’s *t* test (*P*  ≤  0.05)

We next verified if the locus substitution caused changes in abundance of different trichome types and the morphology of type-VI trichomes in adult leaves of the MT-*Sst2* line. We analyzed the gland and internal cavity size of the type-VI glandular trichomes (Fig. 4a, b). There was no significant difference between MT and MT-*Sst2* gland size, which were much smaller than that of LA1777. However, the size of the internal cavity increased in MT-*Sst2* compared to MT. The introgression line also appeared to exhibit an increase in stalk length compared to MT, but still the stalk and the gland cavity were smaller than that of LA1777 type-VI trichomes (Fig. 4a, b).

The majority of glandular trichomes found in MT and MT-*Sst2* was type VI. The density of type-VI trichomes were not altered in MT-*Sst2* compared to MT for both adaxial and abaxial leaf surfaces (Fig. 4c). The wild species showed significantly higher numbers of type-VI trichome on the adaxial and fewer numbers on the abaxial leaf surface.

We also observed that *S. habrochaites* LA1777 had a high density of type-IV trichomes (Fig. 4c). Type-IV trichomes were previously associated with increased production of acylsugars providing resistance to insect pests in wild species (Simmons and Gurr 2005). Type-IV trichomes were however absent on adult leaves of MT and MT-*Sst2*. Type-V is the most abundant non-glandular trichome found on MT and MT-*Sst2*. Interestingly, we found an increased number of (non-glandular) type-V trichomes on both leaf surfaces in MT-*Sst2*. Approximately two-fold more type-V trichomes were observed on the adaxial leaf surface of MT-*Sst2* compared to MT (Fig. 4c).

### Herbivore resistance of the Micro-Tom line with increased amounts of α-santalene, α-bergamotene and β-bergamotene

**Fig. 5:**
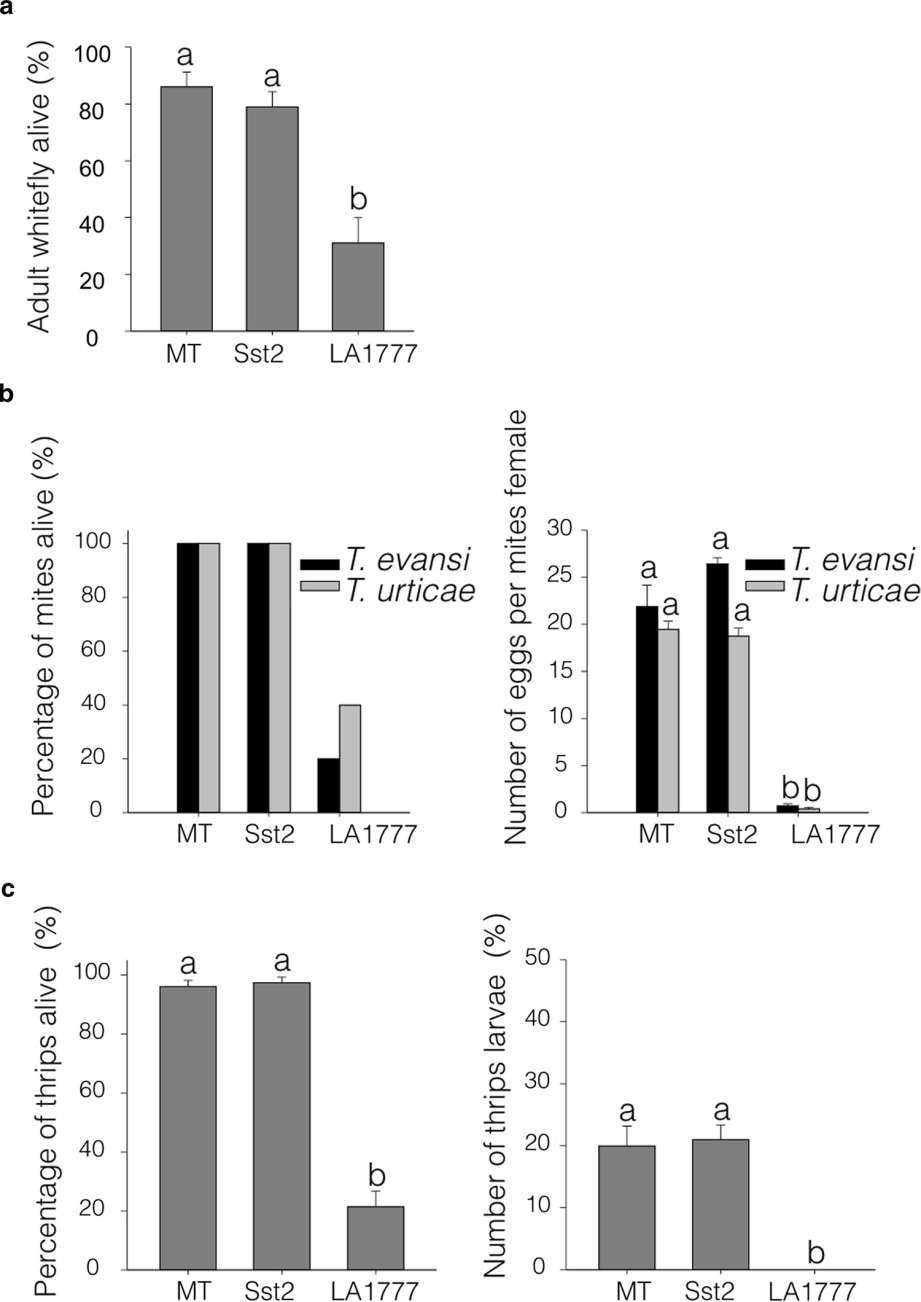
Herbivory tests performed on Micro-Tom (MT), MT-*Sst2* and *Solanum habrochaites* LA1777 genotypes. **a** Percentage of adult whitefly *Bemisia tabaci* alive after 5 days of feeding on leaves. Data are means (±  SE) of five plants, each with two cages. **b** Female spider mite (*Tetranychus evansi* and *Tetranychus urticae*) survival and number of eggs after 2 days of feeding on plants. **c** Percentage of adult thrips alive and number of thrips larvae per female that emerged after 2 days on leaf disks. Bars indicated with different letters were significantly different according to Fisher’s LSD test (*P*  ≤  0.05) after ANOVA

To verify if the increase in plastid-derived sesquiterpenes in type-VI trichomes of MT-*Sst2* would result in improved resistance to piercing-sucking pests, we conducted no-choice bioassays using four relevant pests in tomato. In the whitefly bioassay, survival of whiteflies did not differ between MT-*Sst2* and MT plants, whereas LA1777 displayed a high reduction in the survival of this pest. Almost 80% of the whiteflies survived on MT-*Sst2*, whereas less than 40% survived on the wild tomato species (Fig. 5a).

Next, bioassays using the defense-suppressing spider mite *Tetranychus evansi* and the defense-inducing spider mite *Tetranychus urticae* were performed. Both MT and MT-*Sst2* showed 100% of both spider mites species surviving, whereas on *S. habrochaites* LA1777 only approximately 20% of *T. evansi* and 40% of *T. urticae* survived after 2 days (Fig. 5b). Oviposition rates of both spider mite species were equal on MT-*Sst2* and MT leaf disks, as they were strongly reduced in the wild species (Fig. 5b).

Finally, we conducted a bioassay with western flower thrips (*Frankliniella occidentalis*) comparing adult survival on MT, MT-*Sst2* and the wild species. No significant differences in survival or egg hatching were observed as a result of the *Sst2* introgression (Fig. 5c). Female thrips were still able to oviposit on both genotypes and the larvae hatched from the eggs reaching the larval stage. Conversely, significant reductions in the number of surviving adults and the number of emerged larvae were observed for the wild species.

## Discussion

### *Solanum habrochaites* plastid-derived sesquiterpene synthesis can be transferred to type-VI trichomes of cultivated tomato.

We successfully introgressed the *SsT2* gene cluster responsible for the biosynthesis of plastid-derived sesquiterpene pathway from *S. habrochaites* LA1777 into the genetic model system cv. Micro-Tom (MT) (Fig. 1b). The genes transferred were expressed in tomato type-VI trichomes (Fig. 1d) and resulted in the production of high levels of plastid-derived sesquiterpenes (Fig. 2a–e).

The allelic dosage appeared to have influenced the plastid-derived sesquiterpenes levels produced in type-VI trichomes, however, the correlation between gene expression and metabolite accumulation is not straightforward. The levels of all plastid-derived sesquiterpenes were higher in the homozygous *Sst2*/*Sst2* plants than the hemizygous *sst2/Sst2* (Fig. 3). This can be explained by the presence of just one copy of  both *zFPS* and *SBS* alleles in hemizygous *sst2/Sst2* plants. It can also be the result of the presence of both *SlNDPS1* and *zFPS* in the hemizygous *sst2/Sst2* acting in the same compartment and competing for a limited amount of IPP and DMAPP in type-VI trichomes (Dudareva et al. 2005; Schilmiller et al. 2009; Besser et al. 2009). However, it is unlikely that plastid-derived monoterpene production can limit the substrate for the production of sesquiterpenes in plastids, since the levels of monoterpenes are much lower than the plastid-derived sesquiterpenes (Fig. 3) (Banerjee et al. 2013). In addition, due to differences in substrate affinity, zFPS could use IPP or DMAPP to produce *Z,Z*-FPP more efficiently than SlNDPS1 to produce NPP in the plastids (Sallaud et al. 2009; Kang et al. 2014).

Despite the fact that the introgression line MT-*Sst2* produced higher levels of α-santalene, α-bergamotene and β-bergamotene in type-VI glands (Fig. 2a–e), we did not detect sesquiterpene derivatives in MT-*Sst2* as they are present in the wild species (Fig. 2a–e). The absence of *α*-santalenoic and *α*- and *β*-bergamotenoic acids in the MT-*Sst2* line likely explains the higher levels of α-santalene, α-bergamotene and β-bergamotene, compared to the wild species. In *S. habrochaites* LA1777, α-santalene, α-bergamotene and β-bergamotene are converted to derivatives identified as sesquiterpene carboxylic acids; *α*-santalenoic, and *α*- and *β*-bergamotenoic acids (Coates et al. 1988). Notably, high sesquiterpene levels in MT-S*st2* provide an indirect evidence that α-santalene, α-bergamotene and β-bergamotene are used as precursors for further metabolism into corresponding alcohol and acids derivatives in LA1777 (Frelichowski and Juvik 2005; Besser et al. 2009; Gonzales-Vigil et al. 2012).

The relative transcript levels of *zFPS* and *SBS* were higher in LA1777 compared to the introgressed line (Fig. 1d). Since MT-*Sst2* and LA1777 share the same chromosomal segment comprising the *SsT2* locus, it was expected that they have the same *cis*-regulatory elements controlling the expression of the *zFPS* and *SBS* genes. The lower expression of these genes in MT-*Sst2* may suggest the involvement of a set of yet unknown *trans*-regulatory elements (e.g. transcription factors and other regulatory elements in different chromosomal regions) necessary to increase the expression of the genes present in the *SsT2* locus. Therefore, our results point at a role for additional genetic components that have not been introgressed. It has been shown that poor terpene producing genotypes also have drastically reduced transcript levels for key steps in the terpene biosynthesis pathway (Tissier 2012), suggesting that the transference of additional components enhancing *zFPS* and *SBS* expression could increase the content of sesquiterpenes in MT-*Sst2* even further. So far, only a few transcription factors have been shown to be involved in the regulation of terpene pathways in tomato (Spyropoulou et al. 2014; Xu et al. 2018c), though these were not implicated as positive regulators of *zFPS* or *SBS* specifically.

### Introgression of Sst2 appears to affect type-VI trichome morphology

The MT-*Sst2* line with augmented contents of sesquiterpenes in type-VI trichomes displayed an increased glandular cavity volume (Fig. 4a, b). A possible explanation for this is that the boost in the total amount of terpenes could result in physical pressure against the cavity wall, forcing the internal cavity to inflate like a balloon, as suggested by Ben-Israel et al. (2009). However, the subtle increase in the internal cavity of MT-*Sst2* was not paired with an altered external gland shape. This reinforces the hypothesis that modification in external gland shape depends both on genes related to cell wall remodeling and on synthesis and accumulation of very high levels of compounds into the gland (Bennewitz et al. 2018). Thus, a combination of genes controlling the high flux of metabolites with genes controlling the cell wall remodeling might push the gland to expand creating the characteristic round type-VI trichome of *S. habrochaites*. It is interesting to note that a quantitative trait locus (QTL) analysis of *S. habrochaites* trichome found a major QTL on chromosome 8 (Bennewitz et al. 2018), which coincides with the *SsT2* locus.

Stalk length is one of the morphologic characteristics used to identify the different types of trichomes in *Solanum* (Simmons and Gurr 2005). The slightly increased type-VI trichome stalk length observed in MT-*Sst2* (Fig. 4b) is unlikely the result of the biosynthetic enzymes encoded on the *SsT2* locus. The downregulation of *SlMYC1*, a bHLH transcript factor, results in plants with shorter type-VI trichome stalks (Xu et al. 2018c). Besides, MYCs form transcriptional complexes that regulate defense against herbivores (Erb and Reymond 2019). Since *SlMYC1* (*Solyc08g005050*) is also located on top of chromosome 8, we checked the presence of the wild *ShMYC1* allele inside the region introgressed (Fig. 1c; Figure S1). In general, *S. habrochaites* species exhibit a longer type-VI trichome stalk compared to cultivated tomatoes (Simmons and Gurr 2005; Bergau et al. 2015). The biological role for a higher stalk, or a taller trichome in the wild species is not described, but it is tempting to speculate that though the effect is marginal, the altered stalk length could be the result of replacement of *SlMYC1* with *ShMYC1*.

Furthermore, MT-*Sst2* displayed an increased number of type-V non-glandular trichomes compared to both MT and LA1777 (Fig. 4c). It was shown earlier that there appears to be a negative correlation between densities of type-V trichomes and the glandular type-IV (Vendemiatti et al. 2017). The lower density of type-V trichomes in LA1777 could therefore be related to its high density of type-IV trichomes. The adult leaves of MT-*Sst2* and MT do not have type-IV glandular trichomes (Fig. 4c). The lack of type-IV trichomes in cultivated tomato has been linked to the transition from the juvenility to the adult phase (Vendemiatti et al. 2017). So, the region introgressed in MT-*Sst2* is most likely not involved in heterochrony and/or type-IV trichome development, though we cannot discard the possibility that the region introgressed harbors genes controlling the density of type-V trichomes, as we cannot explain the increased type-V density in the introgression line compared to MT.

### Genetic factors outside the *SsT2* metabolic cluster are required to produce sesquiterpene carboxylic acids (SCA) terpenoids and insect resistance

Even though MT-*Sst2* produced relatively high levels of α-santalene, α-bergamotene and β-bergamotene, compared to *S. habrochaites* LA1777, this did not confer resistance to any of the piercing-sucking tomato pests tested (Fig. 5a–c). Similarly, transgenic tobacco plants emitting santalene and bergamotene did not show an increase in resistance, instead showed augmented attraction to aphids (Yin and Wong 2019). Thus, piercing-sucking pests resistance observed in the wild species could be therefore likely due to the sesquiterpene carboxylic acids (SCAs) derivatives absent in MT-*Sst2*.

We cannot rule out the contribution of type-IV trichomes-derived acylsugars in LA1777 (Kim et al. 2012), but it has been shown before that the presence of the derivatives in LA1777 modulates larval feeding behavior and survival of two lepidopteran pests (Frelichowski and Juvik 2001). Furthermore, the repellent activities of 7-epi-zingiberene derivatives appear to be significantly higher than that for 7-epi-zingiberene (Dawood and Snyder 2020). 9-hydroxy,10,11-epoxy-zingiberene exhibits toxicity against whiteflies, however, 7-epi-zingiberene and 9-hydroxy zingiberene did not affect whitefly survival (Zabel et al. 2021). On the other hand, transgenic *S. lycopersicum* producing 7-epi-zingiberene without the derivatives, did display a clear toxicity phenotype against spider mites and also whitefly repellence (Bleeker et al. 2012). But, it has also been shown that 7-epi-zingiberene does not have effect on the performance, feeding or choice behavior of potato aphid *M. euphorbiae* (Wang et al. 2020). Unitedly, this reveals that the effect of sesquiterpenes and their derivatives can be insect specific.

Up till now, little is known about the enzymes catalyzing the formation of SCAs in *S. habrochaites* LA1777. It can be hypothesized that cytochrome P450 enzymes (which often hydroxylate terpenes) play a role. In *Santalum album* (Santalaceae) santalenes (α-, β- and *epi*-β-santalene) and α-*exo*-bergamotene are further metabolized into sesquiterpene alcohols α-, β-, and *epi*-β-santalol and α-*exo-*bergamotol by a CYP76F cytochrome P450 (Diaz-Chavez et al. 2013). Nevertheless, co-expression of *S. album* santalene/bergamotene oxidase *SaCYP76F39v1* in transgenic tobacco plants did not hydroxylate santalene or bergamotene (Yin and Wong 2019). In *Tanacetum cinerariifolium (*Asteraceae), two oxidation reactions convert trans-chrysanthemol into trans-chrysanthemic acid (Xu et al. 2018b). Using tomato transgenic lines (Xu et al. 2018a), showed that expressing chrysanthemyl diphosphate synthase from *Tanacetum cinerariifolium* together with an alcohol dehydrogenase and aldehyde dehydrogenase from *S. habrochaites* LA1777 were sufficient for the transgenic fruits to produce trans-chrysanthemic acid. For *S. habrochaites* LA2167 a single cytochrome P450 oxidase, ShCYP71D184 present on chromosome 1 (Solyc01g008670), sequentially oxidizes 7‐epi‐zingiberene to 9‐hydroxy‐zingiberene and 9‐hydroxy‐10,11‐epoxyzingiberene derivatives (Zabel et al. 2021).

Although the introgressed region in MT-*Sst2* contains both alcohol dehydrogenases and P450s from *S. habrochaites* LA1777, it seems that the genes required for the conversion of santalene or bergamotene to their alcohols or carboxylic acids derivatives lie outside the metabolic cluster on chromosome 8, as showed before for 7‐epi‐zingiberene in *S. habrochaites* LA2167 (Zabel et al. 2021). Peripheral pathway genes located outside the core metabolic cluster in different chromosomes have been described before for tomato and other species (Nützmann et al. 2016). The fact that we did not detect any sesquiterpene derivative in MT-*Sst2* indicates that the alcohol dehydrogenases and cytochrome P450s introgressed on top of chromosome 8 probably have different functions. A new introgression line using MT-*Sst2* as background for the introduction of the *Sh*P450 can indicate whether the same enzyme is also able to oxidize α-santalene, α-bergamotene, and β-bergamotene to their carboxylic acids as it does for 7‐epi‐zingiberene to their derivatives.

## Conclusion

Altogether, this study demonstrates that the plastid-derived sesquiterpene synthesis from *S. habrochaites* can be transferred to type-VI trichomes of cultivated tomato and this pathway could be also part of the genetic factors necessary to mimic *S. habrochaites* trichome morphology. However, additional genetic components from the wild species should be transferred to acquire piercing-sucking pest resistance in cultivated tomato. The results presented here indicate that some of these genetic components are likely to encode: (i) enzymes that convert α-santalene, α-bergamotene and β-bergamotene into their carboxylic acids, (ii) transcription factors modulating the expression *zFPS* and *SBS* and (iii) enzymes and possible regulatory genes for cell wall remodeling and enlargement of the trichome gland. The introgressed line presented here, in the model system Micro-Tom, can provide for rapid introgression and transgenic manipulation of the additional genetic components involved in sesquiterpene metabolism and type-VI trichome morphology.

## *Author contribution statement*

LEPP and PB planned and designed the research; LEPP designed the introgression. RT, RK, EV and SLI performed experiments and helped to analyze data; SMA and RS helped to analyze data. RT, LEPP and PB wrote the manuscript. All authors read and approved the final manuscript.

## Supplementary Information

Below is the link to the electronic supplementary material.

**Supplementary file 1. **Supplementary data. Table S1. Oligonucleotide sequence used for CAPS markers. Table S2. Oligonucleotide sequence used for quantitative PCR analyses. Fig. S1 Electrophoresis gels showing the positive genetic markers used to differentiate MT-Sst2 from MT. (DOCX 2308 KB).

## Data Availability

The data supporting the findings of this study are available from the corresponding authors, Lázaro E. P. Peres and Petra M. Bleeker, upon request. The genotypes used and described in the paper are also available, upon request, for non-commercial research purposes.
