# Supplementary material for: Introgression of the sesquiterpene biosynthesis from Solanum habrochaites to cultivated tomato offers insights into trichome morphology and arthropod resistance
Source: Planta. 2021 Jun 23;254(1):11. doi: 10.1007/s00425-021-03651-y (PMC8222033; doi:10.1007/s00425-021-03651-y)
Supplement: Supplementary file 1 — Supplementary data. Table S1. Oligonucleotide sequence used for CAPS markers. Table S2. Oligonucleotide sequence used for quantitative PCR analyses. Fig. S1 Electrophoresis gels showing the positive genetic markers used to differentiate MT-Sst2 from MT. (DOCX 2308 KB). [file 425_2021_3651_MOESM1_ESM.docx]

**Table S1.** Oligonucleotide sequence used for CAPS markers.

| **Locus id^1^** | **Forward** | **Reverse** | **Enzyme** | **Fragment** | |
| --- | --- | --- | --- | --- | --- |
|  |  |  |  | **MT** | **LA1777** |
| Solyc08g005020 | TTTGCGTGTACCTTTTGCAG | TCCTCCTCAAAACCCTCTTCCTC | HinfI | 536/512/ 239 | 1048/ 239 |
| Solyc08g005640 | GTTCTCATAGTTCCCACTATGTATCC | GACAAACTACTTTGTTGTCGGAGT | HindIII | 693/155/ 322 | 693/477 |
| Solyc08g006410 | GATCCATTCCTTCCTTGGGCTGTTG | GATGGTATTGTTGGTCCAATTGTC | PacI | 605 | 503/102 |
| Solyc08g007130 | GTCCTTGTGGTGAACTAAGATATCC | GAATATGCCTTCTGCTGATGCTAGG | BglII | 986/490 | 1478 |
| Solyc08g065740 | CGGTGGTCTTAAGGATGAGAAC | CACAACTTCAAAATAGGGTCTC | BanI | 81/286/ 342 | 367/342 |
| Solyc08g076820 | GTACTACTACTCCCTTAGAGCAAC | GTATGCACTAGGGCTCATAATTCG | BspDI | 536/125 | 661 |
| Solyc08g083230 | GATGTGGGTTGTTTTGCAGGA | CCAGACATGGAAAGTGTTAGCGA | TaqI | 618/179/ 106 | 797/106 |

Locus according to Sol Genomics Network database (<http://solgenomics.net/>).

**Table S2.** Oligonucleotide sequence used for quantitative PCR analyses.

| **Primer name** | **Sequence** | **Gene symbol** | **References** |
| --- | --- | --- | --- |
| SBS_QF | GCATTACAGAATGAGTTCGAGG | LOC101250138 | - |
| SBS_QR | CTGATGGTAAATCAAGGCAGC |  |  |
| ZFPS_QF | TTTCGAGGTCTGGAGTAAGAGTG | AHF95235 | - |
| ZFPS_QR | TAGTGCAATCACAAGGTGAAGTC |  |  |
| RCE1_QF | GATTCTCTCTCATCAATCAATTCG | TC153679 | (Van Schie *et al.*, 2007) |
| RCE1_QR | GAACGTAAATGTGCCACCCATA |  |  |

Gene symbol according to National Center for Biotechnology Information database (<https://www.ncbi.nlm.nih.gov/>).


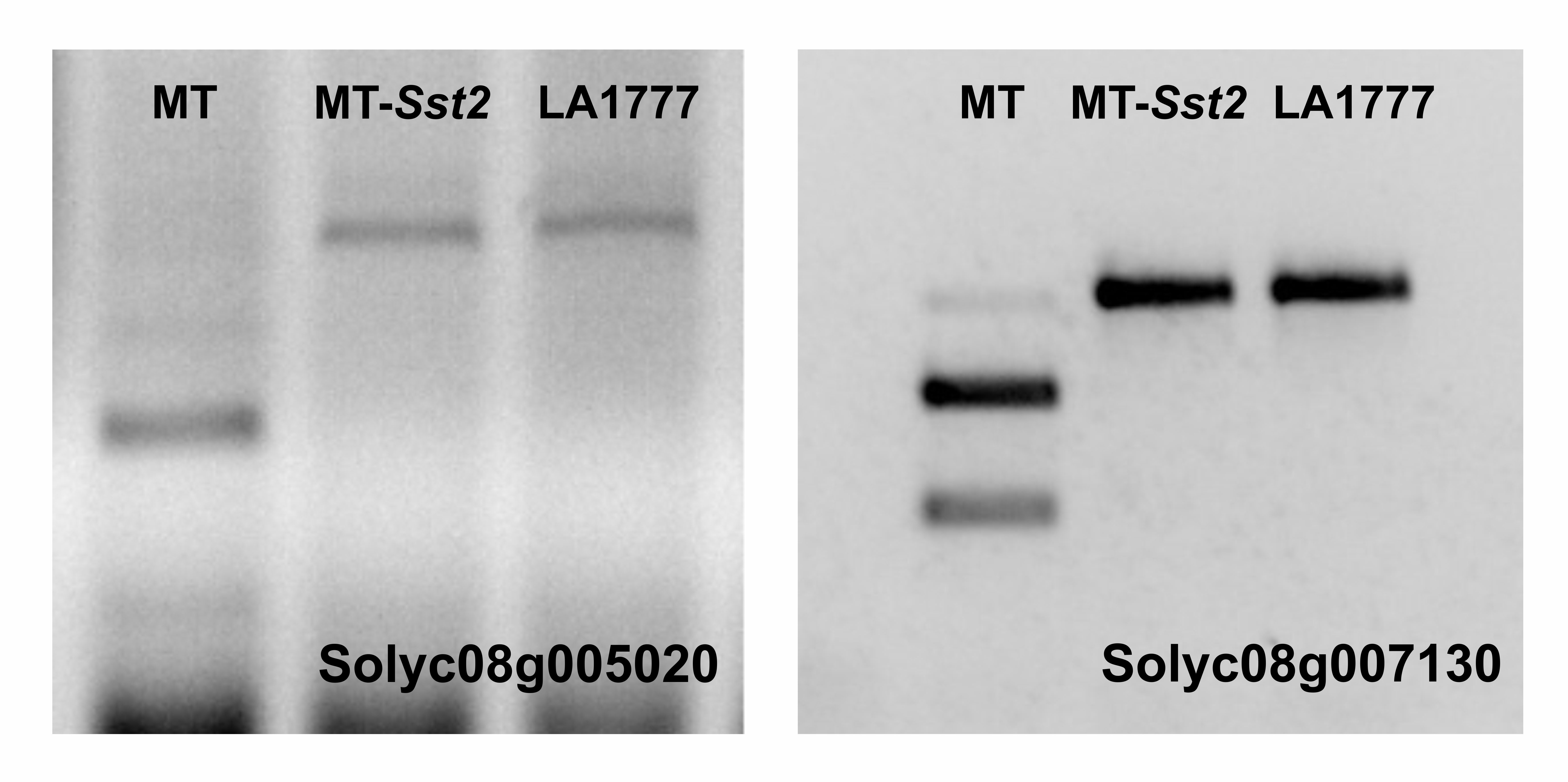


**Fig. S1** Electrophoresis gels showing the positive genetic markers used to differentiate MT-*Sst2* from MT.
